# Microneedles at the Forefront of Next Generation Theranostics

**DOI:** 10.1002/advs.202412140

**Published:** 2025-01-30

**Authors:** Chan Wang, Yuan Yang, Jiaqi Zhang, Hanrui Zhang, Qian Wang, Shengmei Ma, Pengfei Zhao, Zhou Li, Yuxin Liu

**Affiliations:** ^1^ Department of Biomedical Engineering (BME) National University of Singapore Singapore 117583 Singapore; ^2^ Institute for Health Innovation and Technology (iHealthtech) National University of Singapore Singapore 117599 Singapore; ^3^ Beijing Institute of Nanoenergy and Nanosystems Chinese Academy of Sciences Beijing 101400 China; ^4^ State Key Laboratory of Biopharmaceutical Preparation and Delivery Institute of Process Engineering Chinese Academy of Sciences Beijing 100190 China; ^5^ Department of Radiology Beijing Friendship Hospital Capital Medical University No. 95, Yongan Road, Xicheng District Beijing 100050 China; ^6^ School of Nanoscience and Technology University of Chinese Academy of Sciences Beijing 100049 China; ^7^ The N.1 Institute for Health National University of Singapore Singapore 117456 Singapore

**Keywords:** biomarker monitoring, closed‐loop systems, microneedles, theranostics, transdermal drug delivery

## Abstract

Theranostics, combining therapeutic and diagnostic functions, marks a revolutionary advancement in modern medicine, with microneedle technology at its forefront. This review explores the substantial developments and multifaceted applications of microneedles, which have evolved from basic transdermal drug delivery devices to sophisticated diagnostic and therapeutic platforms. Microneedles enhance access to biomarkers via interstitial fluid, enabling real‐time monitoring of physiological conditions, such as glucose and hormone levels, thus facilitating continuous health tracking. The evolution of microneedle design from solid to dissolvable forms broadens their utility from mere drug delivery to complex sensing and therapeutic applications, including insulin delivery for diabetes management, vaccination, and gene therapy. This paper delves into the integration of microneedles with wearable technologies, highlighting their role in closed‐loop systems that combine real‐time monitoring with dynamic, precise therapeutic delivery. By addressing gaps in the literature regarding their integrated diagnostic and treatment capabilities, this review underscores the pivotal role of microneedles in personalizing medicine. It concludes with a visionary perspective on the future trajectory of microneedle technology, emphasizing its potential to revolutionize therapeutic strategies through enhanced efficacy, safety, and patient compliance.

## Graphical abstract

The TOC figure illustrates the revolutionary role of microneedles in modern medicine, showcasing their dual diagnostic and therapeutic functions. On the left, microneedles access interstitial fluid for real‐time monitoring of biomarkers like glucose and hormones. On the right, their therapeutic applications include drug delivery, diabetes management, and gene therapy, highlighting their integration into wearable systems for personalized medicine.

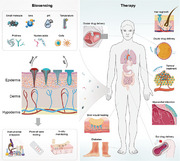

## 1 Introduction

**Figure 1:**
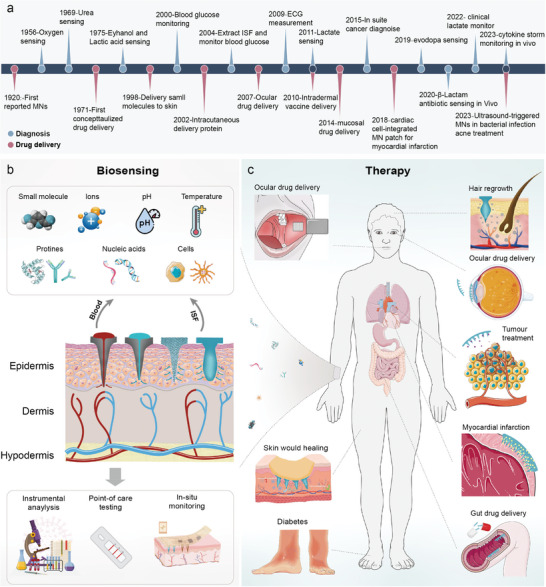
Summary of the research progress of microneedles technology. a) Development history of MNs. b) MNs in biosensing of small molecules, ions, pH, temperature, etc.^[^ ^11^ ^]^ c) MNs in buccal drug delivery,^[^ ^12^ ^]^ hair growth, ocular drug delivery, tumor treatment, wound healing, diabetes, gut drug delivery, and myocardial infarction.

**Figure 2:**
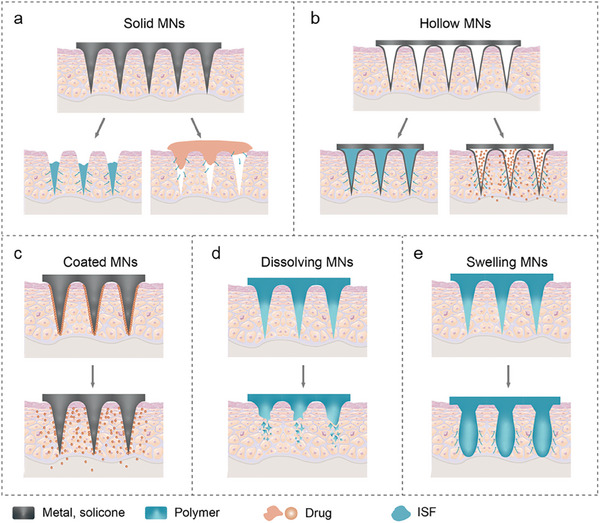
A schematic of five different microneedle types: a) Solid microneedle. b) Hollow microneedle. c) Coated microneedle. d) Dissolving microneedle. e) Hydrogel swelling microneedle.

Theranostics, an amalgamation of therapeutic and diagnostic capabilities, has emerged as a groundbreaking approach in the field of modern medicine.^[^ ^1^ ^]^ At its core, this revolution has been driven by microneedle (MN) technology, which holds promise in both therapeutic and diagnostic realms microneedles have made significant strides in the domain of diagnostics by tapping into interstitial fluid (ISF), thereby accessing a wealth of biomarkers that offer crucial insights into a patient's physiological condition.^[^ ^2^ ^]^ From monitoring glucose levels in diabetic patients to tracking hormone levels, microneedles have demonstrated their potential for continuous, real‐time health monitoring.^[^ ^3^ ^]^ Initially conceived as tools to facilitate transdermal drug delivery by bypassing the skin's stratum corneum, which serves as the primary barrier to absorption, microneedles have undergone a remarkable evolution over the years. The comprehensive overview of microneedle development, including various designs, materials, and applications, is summarized in **Figure** **1**. This figure illustrates the trajectory of microneedle technology from rudimentary designs to sophisticated, integrated systems that offer promise not only in drug delivery but also in personalized medical diagnostics and treatment strategies.^[^ ^4^ ^]^ Their designs, materials, and applications have evolved from solid structures to dissolvable ones, expanding their role from drug delivery to sensing platforms (**Figure** **2**).^[^ ^3^, ^5^ ^]^ This adaptability has allowed microneedles to cater to a wide range of therapeutic agents, from small molecules to macromolecules, with notable applications including insulin delivery for diabetes, vaccines for infectious diseases, and therapeutic nucleic acids for genetic disorders.^[^ ^6^ ^]^ The advantages of microneedles in drug delivery encompass improved bioavailability, reduced side effects, and enhanced patient compliance compared to traditional methods.

The true magic unfolds when therapeutic and diagnostic capabilities converge. Integrated microneedle systems, often complemented by wearable technology, enable the continuous monitoring of biomarkers, based on this real‐time feedback, can deliver precise therapeutic doses.^[^ ^5^, ^7^ ^]^ Such closed‐loop systems hold the promise of a future where treatment regimens are dynamically adjusted in real‐time, embodying the essence of personalized medicine.^[^ ^2^, ^8^ ^]^ In an era where precision, personalization, and patient comfort are paramount, microneedles emerge as beacons of innovation. Their dual role in theranostics ensures that treatment regimens are not mere generalized protocols, but finely tuned responses grounded in real‐time diagnostic feedback.^[^ ^8^, ^9^ ^]^ This approach not only maximizes therapeutic efficacy but also minimizes potential side effects, offering better outcomes and an improved quality of life for patients. In their theragnostic capacity, microneedles exemplify the finest aspects of modern medical innovation by seamlessly blending therapy with diagnostics, promising a future where medicine proactively ensures well‐being.

Existing literature has summarized the advancements in microneedle technology over recent years, particularly focusing on fabrication techniques and materials used;^[^ ^10^ ^]^ there remains a notable gap in comprehensive discussions from the perspective of integrated diagnosis and treatment. This review aims to fill this gap by presenting an updated discussion of the latest developments in microneedle technology. It categorizes the development history of microneedles (Section 1), and extends to exploring the applications of microneedles in biomarker monitoring (Section 2), drug delivery (Section 3), and closed‐loop integrated theranostics systems (Section 4). We conclude with a forward‐looking perspective on the potential future developments and trajectories in the field of microneedle technology (Section 5).

## 2 Microneedles in Diagnostic

While the initial push in microneedle research was largely geared toward drug delivery, there's been a growing interest in leveraging microneedles for diagnostic applications.^[^ ^13^ ^]^ By tapping into the ISF, microneedles can provide insights into physiological and pathological states in real‐time.^[^ ^14^ ^]^ The ISF is a fluid compartment in the body that surrounds cells and provides them with nutrients. It is compositionally like blood plasma, making it an attractive medium for diagnostics. Microneedles offer a less painful and traumatic alternative to traditional blood tests (**Table** **1**).^[^ ^15^ ^]^

**Table 1:** Summary of microneedles in diagnostic.

Biomarkers	Materials	Parameters	Detect range	Long term stability	Ref.
Glucose	chitosan@ N‐Au/PB/CS	length: 1 cm width: 0.5 cm	0.138 µA mM^−1^ in tissue fluid	10 days in 4 °C	[16]
	DA/HA@ PEDOT: PSS	height: 800 µm, pitch: 500 µm, width:200 µm	0‐35 nM	14 days in vivo	[13a]
	PEGDA/MeHA @AuNP	height: 750 µm base: 375 µm	0– 500 mg dL^−1^	NA	[17]
	agarose@Au	height: 2.2 mm, tip diameter: 30 µm, and base diameter: 400 µm	2—13 mM	NA	[3b]
	Cr/Au@Ppy@GOx	NA	1–9 mM 0.66 mM	3–14 days	[18]
	colloidal crystal	width: 400 µm in height: 900 µm	100–400 mg dL^−1^	30 mins	[19]
	Prussian blue@Au	length: 5.2 mm width: 1.6 mm	0.8–24 mM	7 days	[20]
pH	Commercial polymeric adhesive@ Au nanorods	height: 300 µm width: 200 µm	pH: 5–9	NA	[21]
	polyaniline coated PMMA@Au	height: 250 µm tip diameter: 2 µm	pH 4.0 to 8.6	20 days	[22]
	graphite@paraffin oil	tip: 100 µm	pH: 5–9	9 h	[23]
	Dopamine@hyaluronic acid@ PEDOT:PSS	height: 800 µm	pH: 3.5–9	3 h	[24]
alcohol	Pt@PPD/AlOx‐Chit/Nafion	height: 800 µm	0–80 mM	NA	[25]
levodopa	MeHA	height: 600 µm base width: 400 µm	1 µM in skin	NA	[26]
nerve agents	OPH@CP@enzyme	NA	10–200 µm	3 h	[27]
Ions	stainless steel	length: 1.2 cm, base diameter: 440 µm	Ca^2+^: 0.01–100 nM; K^+^: 1–32 nM; Na^+^: 10–160 nM	5 days	[28]
Plasmodium falciparum histidine‐rich protein 2	SU‐8@Au NPs	length: 750 µm diameter: 375 µm	8–32, 8 ng mL^−1^	NA	[29]
IL 6	SU‐8/Au@Carbon nanotube	high: 800 µm; width: 300 µm; tip diameter: 20 µm	0.54; 1–5 pg ml^−1^	5 days in vitro; 24 h in vivo	[30]
	Polystyrene@Fe3O4 NPs@magnetic layer	tip: 4 µm width:300 µm hight: 600 µm	0.33 ng ml^−1^; 2.5 ng ml^−1^–2.5 pg ml^−1^	60 mins	[31]
DNA	CRISPR@CNT	height:600 µm, tip: 30 µm	1.7 fM; 30–30 000 fM	10 days	[6b]
	SU‐8@graphene	height: 800 µm radius: 300 µm top radius:15 µm.	3 × 10^−16^–3 × 10^−13^ m	14 days	[32]
Multiple	MeHA/HA	tip size:10 µm, base diameter: 400 µm, length: 800 µm	phosphate: 0.3–1.8 M , 3.62 µA mM^−1^; uric acid: 50–550 εm, 4.19 nA µM^−1^; creatinine: 50–550 εm, 12.58 nA µM^−1^; urea: 1–16 mM, 44.6 mV decade^−1^	21 days	[33]
	Hyaluronic acid@pH indicator	height:1.3 cm base length: 565.3 µm	pH: 6.0–8.0 uric acid:0–1.5 mMm glucose:0–10 mm temperatures: 36–39 °C	4 days	[34]
	Au/stainless@PMMA	tip diameter: <100 µm diameter: 0.35 mm	Ions: −45.2 mV (−lg[H^+^]^−1^; 30.1 mV (lg[K^+^]^−1^, 30.2 mV (lg[Na^+^]^−1^, 21.0 ± 1.6 mV (lg[Ca^2+^]^−1^ ROS: 0–100 mM; glucose: 0–20 mM); Uric acid: 0–0.8 mM	NA	[35]

**Figure 3:**
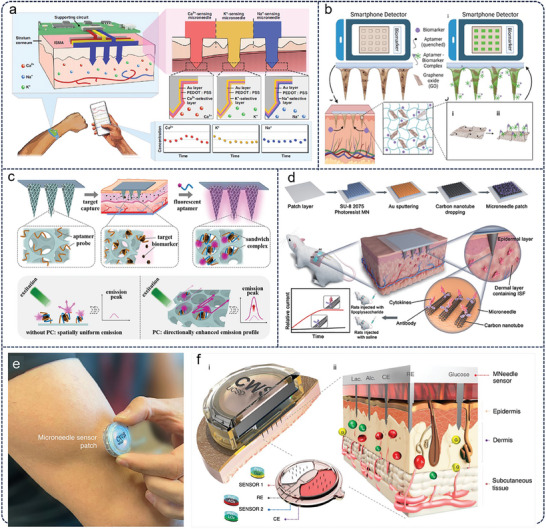
Microneedles in diagnostic. a) 3D assembled MNs ion sensor for physiological ion fluctuations monitoring.^[^ ^40^ ^]^ Copyright 2023, The Author(s) b) Hydrogel MNs for on‐site detection of small molecules and proteins.^[^ ^42^ ^]^ Copyright 2016 Published by Elsevier Inc. c) Inverse opal MN arrays for fluorescence‐enhanced screening of ISF biomarkers.^[^ ^41b^ ^]^ Copyright 2022 Published by Elsevier Ltd d) Wearable, non‐invasive MN patch to predict cytokine storm.^[^ ^8a^ ^]^ Copyright 2023 Wiley‐VCH GmbH e,f) Integrated wearable microneedle array for the continuous monitoring of glucose, lactate, and alcohol in ISF.^[^ ^44^ ^]^ Copyright 2022, The Author(s), under exclusive license to Springer Nature Limited.

The ion concentrations in blood are important reference indicators related to many diseases. Imbalances can lead to severe health issues, such as dehydration, heart arrhythmias, or even seizures. Electrolytes, including sodium (Na^+^), potassium (K^+^), calcium (Ca^2+^), and chloride (Cl^−^), are essential for a multitude of physiological functions.^[^ ^36^ ^]^ Microneedle‐based sensors have been researched to monitor these ions, especially in patients with conditions like kidney disease or heart problems. The initial studies of microneedles in electrolyte monitoring aimed to validate the concept of obtaining accurate electrolyte measurements from ISF using microneedles.^[^ ^37^ ^]^ Electrodes functionalized with ion‐specific coating were integrated with microneedles to selectively detect target electrolytes. Solid microneedles were used to create microchannels for ISF extraction, which is then analyzed using external sensors. Hollow microneedles facilitate continuous ISF sampling, channeling the fluid to integrated ion‐sensitive sensors.^[^ ^38^ ^]^ Hydrogel microneedles swell upon insertion, absorbing ISF and its constituent electrolytes for subsequent analysis.^[^ ^39^ ^]^ Ion‐selective electrodes use membranes that are selectively permeable to specific ions, providing measurements based on the electrochemical potential. Xie et al.^[^ ^40^ ^]^ developed a microneedle sensing‐array integrated system for sufficiently sensitive detecting real‐time changes in Ca^2+^, K^+^, and Na^+^ concentrations with good detection performance (**Figure** **3**a). The integrated microneedle sensor array system includes three interconnected modules: the ion sensing microneedle array (ISMA), a printed circuit board for recording and control, and a mobile application for real‐time monitoring. The ISMA uses three parallel electrodes to simultaneously detect Ca^2^⁺, K⁺, and Na⁺, with ion selective membrane ensuring only target ions reach the electrode surface to realize the target‐specific recognition. At the same time, the researchers demonstrated through in vivo experimental results that the ion‐sensing microneedle array can monitor the concentrations of Ca^2^⁺, K⁺, and Na⁺ in real time, confirming the potential clinical applicability of the ion‐sensing microneedle array and providing recommendations for clinical treatment. At the same time, the in vivo experimental results demonstrated that the ion‐sensing microneedle array can monitor the concentrations of Ca^2^⁺, K⁺, and Na⁺ in real time, confirming the potential clinical applicability of the ion‐sensing microneedle array and providing suggestions for clinical treatment.

Metabolic biomarkers in ISF indicate human physiological processes and disease states and are widely used for medical diagnosis and health monitoring.^[^ ^41^ ^]^ Simultaneous monitoring of multiple biomarkers is difficult because it often involves multiple enzymes and complex preparation processes. Poudineh et al.^[^ ^42^ ^]^ developed an optical hydrogel microneedle (HMN) based on nucleic acid probes, capable of simultaneous capture and detection analysis (Figure 3b). The HMN uses methyl methacrylate (MeHA) as its hydrogel structure and incorporates graphene oxide‐nucleic acid optical sensing elements. Fluorescently modified nucleic acids (NA) are fixed on graphene oxide nanosheets (GO), which act as quenchers and fixatives. Single‐stranded NA binds strongly to GO, quenching fluorescence in the absence of a target. When the target is present, NA binds to it, changing conformation and restoring fluorescence to generate a signal. To enable visualization, smartphone‐based fluorescence image acquisition software was developed. The analytes glucose, uric acid, serotonin, and insulin were released by using four different aptamers, showcasing the versatility of the HMN‐GO‐NA system.

Beyond traditional targets like glucose or hormones, researchers are interested in a broader range of metabolites and proteins.^[^ ^43^ ^]^ Monitoring these molecules can provide insights into metabolic health, disease progression, and therapeutic responses. Monitoring cholesterol in ISF can offer insights into lipid metabolism and cardiovascular health. Levels of certain amino acids can be indicators of metabolic disorders or dietary imbalances. Elevated uric acid levels in ISF can correlate with conditions like gout or kidney stones. Cytokines are small proteins critical for cell signaling. Monitoring cytokine levels can be indicative of inflammatory responses or immune system activity. Protein biomarkers like troponin can indicate heart attack, while others like prostate‐specific antigen are used in cancer screening. Monitoring levels of administered therapeutic proteins can guide dosing and treatment strategies. The exploration of microneedles for monitoring a wider range of metabolites and proteins exemplifies the potential breadth of this technology in personalized medicine and theranostics. Continuous, real‐time data on these molecules can help tailor treatments, predict disease progression, and better understand individual metabolic and physiological responses. Zhao et al.^[^ ^41b^ ^]^ reported a novel inverse opal microneedle array for in situ extraction and fluorescence‐enhanced detection of target biomarkers in skin ISF (Figure 3c). The microneedle array was prepared by applying a negative mold of resin‐replicated filled colloidal crystals with unique porous structures and photonic band gap properties. These properties enable the inverse opal microneedle array to achieve non‐invasive skin ISF sampling and provide enhanced fluorescence signal intensity for specific biological probes, thereby improving detection sensitivity. The researchers further validated the ability of the microneedle array to efficiently and sensitively extract and detect lipopolysaccharide in the ISF of infected rats, showing that the inverse opal microneedle array has great potential value in non‐invasive clinical diagnosis and other related biomedical applications.

Fang et al.^[^ ^8a^ ^]^ introduced a wearable microneedle patch based on a functionalized carbon nanotube bio‐interface for real‐time monitoring and early warning of cytokine storms in vivo (Figure 3d). The microneedle patch achieves high‐sensitivity detection of cytokines through electrochemical analysis, with a minimum detection limit of 0.54 pg mL^−1^, high specificity and 5‐day stability, and a coefficient of variation of 4.0%. The system responds rapidly to the increase of cytokines, taking only 1 to 4 h. The wearable device has achieved real‐time capture and monitoring of protein markers in ISF for the first time, providing a powerful tool for early diagnosis, disease assessment, and prognosis of inflammatory diseases such as sepsis. The study results show that the microneedle patch has important application value in clinical and scientific research. Wang et. al.^[^ ^44^ ^]^ detailed the development and evaluation of a fully integrated wearable microneedle array for continuous monitoring of multiple biomarkers in ISF (Figure 3e,f). The device enables real‐time, wireless sensing of biomarkers such as glucose, lactate, and alcohol, and is designed for user comfort, offering a pain‐free and user‐friendly experience. The system comprises reusable electronics coupled with a disposable microneedle array, with data transmitted to a custom smartphone application for visualization. Their on‐body trials demonstrated a strong correlation between the device's measurements and traditional blood or breath tests, highlighting its potential as a valuable tool for personalized health monitoring and early disease diagnosis. The exploration of microneedles for monitoring a wider range of metabolites and proteins exemplifies the potential breadth of this technology in personalized medicine and diagnostics. Continuous, real‐time data on these molecules can help tailor treatments, predict disease progression, and better understand individual metabolic and physiological responses.

In conclusion, microneedles for diagnostic applications have made significant advancements. However, several challenges remain, including ensuring biocompatibility and safety to prevent immune reactions, overcoming the complexity of manufacturing intricate microneedle structures, maintaining stability and reliability in diverse real‐world conditions, and achieving seamless data processing and system integration for closed‐loop health monitoring. Despite these obstacles, microneedles hold great promise for the future of healthcare sensing applications.

## 3 MNs for Drug Delivery

Invasive administration, such as traditional needle injections, is commonly associated with discomfort and suboptimal patient compliance. Alternatively, non‐invasive routes, such as oral administration, offer the convenience of self‐administration for patients. Nevertheless, the physiological tissue barrier poses a limitation, necessitating the ingestion of exceedingly high doses of certain drugs, which subsequently translates into heightened medical expenses and the risk of adverse side effects. Microneedles, featuring optimized physical attributes, emerge as a promising minimally invasive platform for drug delivery.^[^ ^80^ ^]^ This section discusses the utilization of microneedles to overcome physiological barriers to the delivery of different kinds of goods (such as small molecule drugs, nucleic acids and proteins, fluids, and cells) (**Table** **2**) to tissues such as the skin, stomach, heart, and eyes.

**Table 2:** Types of drugs and agents delivered via microneedles.

Type	Example	Function	Ref.
Small molecule	Sodium valproate	Hair regeneration	[45]
	Lidocaine	Analgesia	[46]
	Metformin	Diabetes treatment	[79]
	Dexamethasone	Anti‐inflammatory	[47]
	Doxorubicin	Anticancer	[48]
Nucleic acid	DNA vaccine	Vaccination, cancer immunotherapy	[49]
	DNA aptamer	Block protein	[50]
Protein or peptide	Insulin	Diabetes treatment	[51]
	Virus‐related antigen	Vaccination	[52]
	Antibody	Disease treatment	[53, 54]
	Collagen	Cosmetics and wound healing	[55]
	Tumor‐related antigen	Cancer immunotherapy	[56]
Cell	CAR T cell	Anti‐cancer	[57]

### 3.1 Types of Drugs and Agents Delivered via Microneedles

#### 3.1.1 Small Molecule

Small drug molecules (>500 Da) can passively penetrate the skin, but for high‐dose medications, passive diffusion often fails to reach therapeutic levels. MNs create micropores that significantly enhance the delivery of small molecules, improving therapeutic efficiency. Hydrophilic drugs dissolve easily in the polymer solutions used for microneedles, allowing effective drug loading.^[^ ^45^, ^48^, ^58^ ^]^ For lipophilic drugs, water‐soluble polymers in microneedles limit drug solubility and loading. This challenge can be addressed by enhancing interactions between drugs and polymers, such as ionic bonds, hydrophobic interaction, and host–guest interaction.^[^ ^59^ ^]^ Researchers^[^ ^60^ ^]^ have increased lipophilic drug loading by using HP‐β‐cyclodextrin,^[^ ^61^ ^]^ which encapsulates drugs like diclofenac sodium and triamcinolone acetonide, significantly improving drug delivery efficiency.

#### 3.1.2 Nucleic Acid

Microneedles enhance the delivery of nucleic acid vaccines by protecting them from degradation and enabling sustained immune stimulation.^[^ ^49a^ ^]^ DeMuth et al. improved immune responses by layering plasmid DNA and immune‐stimulating RNA on microneedles.^[^ ^49b^ ^]^ Beyond vaccination, microneedles deliver therapeutic genetic drugs, such as VEGF‐inhibiting DNA aptamers.^[^ ^50^ ^]^

#### 3.1.3 Protein

For proteins, there are some problems in their clinical application, such as high molecular weight, poor stability in vitro and in vivo, variability or degradation inactivation. As a new preparation between subcutaneous injection and transdermal patch, microneedles can break through the stratum corneum barrier to restrict macromolecular drugs and improve skin penetration, and be freeze‐dried to maintain biological activity and facilitate transportation and preservation. Lin et al.^[^ ^62^ ^]^ successfully prepared thymus pentapeptide‐dissolved microneedles by a two‐step method and maintained structural integrity and biological activity after drug incorporation. Notably, in vivo pharmacodynamic results showed that compared with intravenous injection of thymus pentapeptide, microneedles could achieve a comparable immunomodulatory effect. In addition, microneedles have been widely used for transdermal delivery of insulin. Chen et al.^[^ ^51^ ^]^ designed and developed a combined microneedle patch with multiple drug release characteristics, which can closely align with daily postprandial insulin requirements and effectively reduce daily blood sugar fluctuations.

Microneedles are also widely used to deliver immune checkpoint inhibitors that block or reverse tumor immunosuppressive microenvironments. Ye et al.^[^ ^54^ ^]^ developed HA microneedles for co‐delivery of PD‐1 antibody and 1‐methyltryptophan (1‐MT) to treat melanoma, enhancing T cell‐mediated killing and reducing regulatory T cells (Tregs). Wang et al.^[^ ^63^ ^]^ introduced self‐degrading microneedles with pH‐responsive nanoparticles that release PD‐1 antibodies in acidic environments, improving delivery precision and efficacy.

#### 3.1.4 Cells

Microneedles offer a promising platform for cell therapy, enhancing the delivery of living cells while maintaining viability and mechanical strength. Chang et al.^[^ ^64^ ^]^ developed frozen microneedles that preserve cell longevity and enable effective dermal delivery, simplifying preparation and reducing contamination risks. In immunotherapy, Chen et al.^[^ ^57^ ^]^ designed porous microneedles for CAR‐T cell delivery to solid tumors, improving cell distribution and therapeutic efficacy. For tissue regeneration, Lee et al.^[^ ^65^ ^]^ created hybrid microneedles for mesenchymal stem cell delivery, promoting localized wound healing and minimizing immune reactions. These advancements highlight microneedles as efficient, minimally invasive tools for cell‐based therapies.

### 3.2 Different Application Sites

#### 3.2.1 Skin

As a drug delivery carrier, microneedles have been applied to many sites including skin, buccal, intestine, ocular tissue, and heart, etc. For skin administration, microneedles are a new type of transdermal drug delivery system whose main principle is to penetrate the skin layer, thereby forming micron‐sized channels that deliver drugs directly into the epidermis or upper dermis area, where drugs can be directly circulated throughout the body without facing a barrier. The system has been widely used in the percutaneous delivery of oligonucleotides, vaccines, growth factors, insulin, etc., and has been applied in the treatment of tumors,^[^ ^66^ ^]^ tissue repair,^[^ ^67^ ^]^ skin diseases,^[^ ^68^ ^]^ and diabetes.^[^ ^69^ ^]^

#### 3.2.2 Oral Cavity

**Figure 4:**
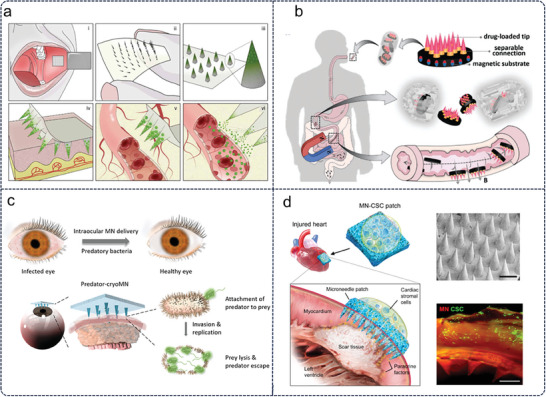
Microneedles are applied to diverse body parts. a) Schematic illustration of the buccal administration with microneedles.^[^ ^71^ ^]^ Copyright 2021, Publisher, AAAS. b) Schematic illustration of the intestine administration with microneedles controlled by a magnetic field.^[^ ^73^ ^]^ Copyright 2021, Publisher, Wiley‐VCH. c) Schematic illustration of microneedles delivery bacterivorous into ocular tissue.^[^ ^75^ ^]^ Copyright 2021 The Authors. Advanced Science published by Wiley‐VCH GmbH. d) Schematic illustration of microneedles delivery of stromal cells into the heart.^[^ ^76^ ^]^ Copyright 2018, Publisher, AAAS.

For the oral cavity, microneedles for drug delivery could avoid problems such as poor drug retention caused by continuous saliva secretion, and the movement of the tongue during chewing severely hindering the absorption of drugs. Zhang et al.^[^ ^70^ ^]^ developed biodegradable methacrylate gelatin (GelMA) microneedles that deliver antibiotics and cytokines into local gum tissue for immune regulation and tissue regeneration. Furthermore, due to the abundance of sensory nerves and microorganisms in the oral mucosal cavity, it is essential to systematically study the safety and painlessness of microneedling in oral applications. In a clinical trial involving 100 healthy volunteers, 95% of the volunteers preferred microneedle oral injections over traditional injections (**Figure** **4**a). These findings encourage the prospect of developing a broader range of microneedle‐based oral drug delivery systems.^[^ ^71^ ^]^

#### 3.2.3 Intestine

Oral administration offers convenience and high patient compliance, but delivering macromolecular drugs (proteins, peptides) remains challenging due to gastrointestinal (GI) degradation. Microneedle technology presents a novel solution by forming micron‐scale channels to deliver drugs directly into GI tissues. However, issues such as GI peristalsis and digestive fluid erosion can hinder efficacy. To address this, Robert Langer and Giovanni Traverso^[^ ^72^ ^]^ developed a dynamic omnidirectional adhesive microneedle system that anchors tablets to the stomach lining, enhancing drug absorption and bioavailability.

The researchers developed a magnetically controlled microneedle robot (Figure 4b). The tip of the microneedle robot can be positioned on the wall of the small intestine under a specific magnetic field, overcoming obstacles to insert tissue and deliver encapsulated drugs. It has been proved in a porcine model that oral insulin by a microneedle robot can effectively regulate blood glucose.^[^ ^73^ ^]^ Additionally, a microneedle robot with a magnetic substrate and degradable tip was developed for targeted small intestine delivery. The system allows precise positioning and sustained drug release, demonstrated by effective insulin delivery in pigs. While promising, further testing in larger animals and human study is needed. Factors like mealtime fluctuations and GI conditions also necessitate improved biomaterials and device designs to ensure stability and predictable drug release. Microneedles hold the potential to revolutionize drug delivery in the gastrointestinal tract, addressing drug non‐compliance and expanding treatment options for macromolecular therapies.

#### 3.2.4 Ocular Tissue

Microneedles, which can deliver drugs to the anterior and posterior segments of the eye, have received extensive attention in the application of ocular diseases. When applied to the anterior segment, microneedles can penetrate the tear film and cornea to directly increase the amount of drug penetration, while when applied to the periocular administration, drugs can be delivered to the posterior segment of the eye by inserting the sclera or uvea. Microneedles are less invasive than eye injections, reducing the risk of pain and infection.^[^ ^74^ ^]^ Xu et al. prepared frozen microneedles loaded with bacteria for the keratitis treatment.^[^ ^75^ ^]^ In conclusion, ocular microneedles have obvious advantages, can significantly improve patient tolerance, reduce adverse reactions, improve bioavailability, and are expected to achieve industrial production, bringing light to patients with visual impairment worldwide.^[^ ^75^ ^]^

#### 3.2.5 Heart

In the treatment of heart disease, intracardiac injection is a direct and effective way to deliver drugs to the heart. However, the amount of injection must be very limited, and there are problems with injection site damage and leakage when the heart beats. Microneedles can puncture the epicardial membrane for local drug delivery and can provide mechanical support, which has aroused great interest in the field of cardiovascular medicine. Myocardial infarction is a common cardiovascular disease caused by cardiac cell death caused by myocardial ischemia. The main disease features of myocardial infarction include fibrosis formation, ventricular remodeling, and contractile defects. Junnan Tang et al. designed microneedle angiogenesis and to heart stromal cells to promote heart repair (Figure 4d).^[^ ^76^ ^]^ The microneedles successfully penetrated the pericardium and penetrated the myocardium, allowing the release of regenerative factors secreted by CSCs into the damaged myocardium, significantly promoting cardiomyocyte proliferation and angiogenesis, and reducing cell apoptosis.

The preparation of microneedle patches for cardiac gene transfer is also a promising method for the treatment of myocardial diseases. In contrast to direct local intra‐myocardial injection (in which gene‐expressing cells are restricted to the injection site), uniform distribution of transfected genes was achieved using microneedles carrying adeno‐associated virus (AAV)‐9 (MNAAV).^[^ ^77^ ^]^ Therefore, MN‐AAV encoding the VEGF gene showed more specificity in the treatment of rats with myocardial infarction than direct intramuscular injection.

### 3.3 MNs for Stimulus‐Response Drug Delivery

In recent years, the application of stimulus‐responsive materials to microneedle drug delivery systems has gained widespread attention. Stimulus‐responsive microneedles can regulate the drug release rate by sensing endogenous physiological signals or exogenous physical stimuli to achieve on‐demand release. The release behavior of microneedles varies with the intensity of external stimulation, enabling the release of a certain dose of the drug at a specific time and space, thereby improving the therapeutic effect of the drug and reducing potential side effects of the drug.

#### 3.3.1 Light Response

Researchers have applied photo‐responsive materials in microneedle drug delivery, where microneedles respond to specific wavelengths of light, such as UV, visible, and near‐infrared light, to control drug release. Near‐infrared light‐responsive microneedles have gained widespread attention because NIR light can penetrate human skin tissue well without causing damage to skin tissue. Zhang et al.^[^ ^78^ ^]^ prepared a NIR light‐triggered drug delivery microneedle patch with a tip consisting of lauric acid (LA) and polycaprolactone (PCL), encapsulated with both the therapeutic diabetes drug metformin and NIR light‐responsive Cu_7_S_4_ nanocrystals. Under the irradiation of NIR light, the Cu_7_S_4_ nanocrystals convert light energy into heat energy through photothermal conversion, causing the LA/PCL to melt, thereby releasing the encapsulated metformin. When the light‐responsive microneedle encapsulates 1 mg of metformin and 0.1 wt.% of Cu_7_S_4_ nanoparticles, it can maintain normal blood glucose levels for 4.5 h. This NIR light‐responsive microneedle delivery system allows on‐demand drug delivery for effective glycemic control.

**Figure 5:**
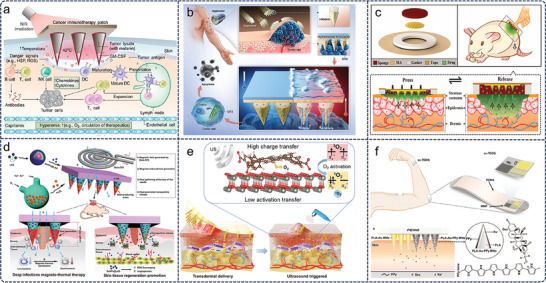
MNs for stimulus‐response drug delivery. a) Light‐responsive microneedle patch for cancer immunotherapy.^[^ ^79^ ^]^ Copyright 2017, Publisher, AAAS. b) Hydrothermally responsive multi‐round capturable microneedle (HRMAM) system.^[^ ^81^ ^]^ Copyright 2023, Publisher, Elsevier. c) Stress‐driven microneedle patch for controlled delivery of liquid macromolecules.^[^ ^82^ ^]^ Reproduced with permission. d) Wireless magnetically responsive microneedle for controlled drug release.^[^ ^83^ ^]^ Copyright 2019, Publisher, Elsevier. e) Ultrasound‐responsive nanoparticles for effective acne treatment.^[^ ^85^ ^]^ Copyright 2023, Publisher, AAAS. f) Self‐powered controllable transdermal drug delivery system.^[^ ^87^ ^]^ Copyright 2021, Publisher, Wiley‐VCH.

In addition to being applied to drug control, light‐responsive microneedles can also exert synergistic effects of chemotherapy and photothermal therapy for the treatment of oncological diseases. Ye et al.^[^ ^79^ ^]^ proposed a melanin‐mediated light‐responsive microneedle patch for cancer immunotherapy, where melanoma lysates containing melanin are encapsulated in microneedles (**Figure** **5**a). Under near‐infrared light, the melanin‐mediated microneedle generated heat to promote the release of the lysate. Simultaneously exerting a bio‐thermal effect, the elevated temperature promotes blood and lymph flow, activates, and induces immune cells, enhances T‐cell activity, and promotes an anti‐tumor immune response. This increased immune response amplifies the body's ability to respond to lysates and better prevent melanoma invasion. After treatment with NIR light‐responsive microneedles, tumor‐bearing rats survived in good health and 87% of the rats no longer had tumor cells in their bodies.

#### 3.3.2 Temperature Response

It is generally prepared from a temperature‐sensitive material, and after the microneedle is pierced into the skin, the temperature of the skin surface is adjusted to make the microneedle substrate material undergo a phase change from a solid to a liquid state to achieve controlled release of the encapsulated drug. Zhang et al.^[^ ^80^ ^]^ proposed a thermosensitive microneedle array consisting of a PCL tip and a PVA/PVP substrate. After microneedle action to the skin, the PCL tip can be easily separated from the substrate and implanted in the skin. Once the temperature is heated to 50 °C, the PCL tip changes from a solid to a liquid state, releasing the loaded metformin, thus enabling effective control of drug release by regulating the temperature change. Compared with subcutaneous injection, the heat‐responsive microneedle can release metformin for a longer period in a diabetic rat model experiment. When microneedles were encapsulated with 16 mg of metformin, normal blood glucose levels could be effectively maintained for 3.5 h. However, since the transition temperature of PCL (≈50 °C) is very close to the endurance limit of the human body, thermal damage can occur if the skin is exposed to high temperatures for a long time. Therefore, to reduce side effects, the design of microneedles must be further optimized. A hydrothermally responsive multi‐round acquirable microneedle (HRMAM) system is further reported, which is inspired by a self‐heating hot pot hydrothermal system and intelligent bionic spine structure (Figure 5b).^[^ ^81^ ^]^ The system is equipped with Docetaxel (DTX) as an anti‐tumor drug and the main component is Polycaprolactone (PCL), which achieves the synergistic effect of hydrothermal chemotherapy by integrating a hydrothermal reaction formula like a self‐heating hotpot heating package into the microneedle substrate.

#### 3.3.3 Stress Response

Stress stimulation can also be used to control drug release. Jiang et al.^[^ ^82^ ^]^ prepared a stress‐driven microneedle patch for controlled delivery of liquid macromolecules (Figure 5c). The patch is a transdermal drug delivery patch with a solid microneedle integrated into the reservoir, and the drug release is controlled by a press‐release action. When pressure is applied to the patch, the solid microneedle spikes are pressed to penetrate the skin stratum corneum and create microchannels on the skin surface, and when the pressure is removed, the solid microneedles are detached from the skin and the drug rapidly enter the skin tissue through the microchannels for disease treatment. Diabetic rats showed a slow decrease in blood glucose levels and maintained a normoglycemic state for 7.7 h after treatment with a drug‐loaded 5 IU insulin microneedle patch, demonstrating the good insulin transdermal delivery capability of the stress‐driven microneedle patch. This method provides patients with an on‐demand recurrent dosing method controlled autonomously at home. However, this microneedle delivery system has some pending issues, such as how to reduce insulin residues in the reserve layer and how to determine the quantitative relationship between pressure and dosage.

In addition, Di et al.^[^ ^69a^ ^]^ investigated a wearable tensile strain‐triggered drug delivery device consisting of an elastomer containing drug‐laden nanoparticles and hydrogel microneedles. The device was attached to human skin, the elastomer was strained by force, and the internal structure was stretched and compressed, thus facilitating the entry of its encapsulated drug into human skin tissue through hydrogel microneedles. When the insulin dose contained in the nanoparticles is 30 mg, the blood glucose level of mice rapidly decreases to a normoglycemic state within 30 min after each stretching cycle. This microneedle drug delivery system can well achieve the on‐demand release of insulin and effectively regulate blood glucose levels.

#### 3.3.4 Magnetic Response

Jayaneththi et al.^[^ ^83^ ^]^ investigated a wireless magnetically responsive microneedle for controlled drug release. Controlled release of drug through hollow microneedles was achieved by controlled deformation of magnetic polymer composite by a magnetic field. The system is capable of drug dispensing and dose sensing without the need for electronic devices or batteries. Experimental tests have shown that this battery‐free device exhibits excellent controlled drug release properties by effectively piercing the microneedle into the skin and injecting the drug into the tissue under the action of a magnetic field. Yang et al. developed a magneto‐thermal response double‐layer microneedle (Fe‐Se‐HA‐MNs) consisting of functionalized hyaluronic acid (HA), ferric oxide (Fe_3_O_4_), and micellar‐protected selenium nanoparticles (SeNPs@LAS) based on a self‐designed dish‐shaped electromagnetic field device (Figure 5d).^[^ ^84^ ^]^ The electromagnetic field produced by disk ZVS has little attenuation in intensity in living tissue. Finite element simulation shows that the electric field strength and electromagnetic loss are concentrated at the tip of Fe‐Se‐HA‐MNs. MNs can pierce hard scars, penetrate bacterial biofilms, and perform effective magnetothermal conversion for deep high‐temperature sterilization. Subsequently, excess hyaluronidase in diabetic wounds can gradually degrade Fe‐Se‐HA‐MNs to release SeNPs, thereby reducing reactive oxygen species to regulate wound REDOX homeostasis. At the same time, SeNPs are beneficial to angiogenesis, promote blood vessel formation, and promote wound repair. Therefore, Fe‐Se‐HA‐MNs can achieve a variety of functions, such as magnetothermal disinfection, deep noninvasive tissue penetration, anti‐inflammatory, and pro‐angiogenesis, which shows great potential as an adjoint treatment for infectious diabetic wounds.

#### 3.3.5 Ultrasonic Response

Kelvin W. K. Yeung et al.^[^ ^85^ ^]^ reported a sodium hyaluronate microneedle patch that mediates transdermal delivery of ultrasound‐responsive nanoparticles for effective acne treatment (Figure 5e). The ZnTCPP@ZnO composite structure material was synthesized and loaded into the microneedles synthesized from sodium hyaluronate. The interface effect between composites can greatly improve the acoustic catalytic performance and effectively reduce the energy required for oxygen activation. Under ultrasound, oxygen obtains more electrons through high interfacial charge transfer and changes from the ground state to the excited state, thus rapidly producing many reactive oxygens, and under 15 min of ultrasonic irradiation, the reactive oxygen‐mediated killing rate of Bacillus acne was 99.73%, and the levels of acne‐related factors (including tumor necrosis factor‐α, interleukin, and matrix metalloproteinases) were reduced.

#### 3.3.6 Electro‐Responsive

With the advent of the smart era, wearable smart medical electronics have achieved rapid development. Due to the easy implementation of electrical stimulation and the diversified modes of output electrical signals, electrically responsive microneedle drug delivery systems have become another research hotspot for responsive microneedle drug delivery systems. To achieve controlled drug release from microneedles, drugs can be encapsulated in electrically responsive microneedles, and the drug release rate can be regulated by electrical stimulation. Iontophoresis is a method of adjusting the process of drug penetration in the skin by varying the applied electric current. This method requires applying the drug to an electrode with an opposite charge to the drug, using the principle of mutual repulsion of the same charge to enhance the drug penetration in the skin. Li et al.^[^ ^86^ ^]^ reported a solid polymer‐based ion‐conductive porous microneedle that combines ion introduction technology with a microneedle drug delivery system to enhance drug transdermal rates via direct current. This microneedle can penetrate the high‐impedance stratum corneum, and under the effect of electrical stimulation, the drug is rapidly released into the skin through the porous microneedle. Conductive polymers are a class of polymers that form conjugated π‐bonds through chemical or electrochemical doping and thus transform from insulator to conductor. Conductive polymers mainly include polypyrrole (PPy), polyaniline, polythiophene, and their derivatives, which have the advantages of good electrical conductivity, simple synthesis, processing flexibility, low density, and good mechanical compatibility. Yang et al.^[^ ^87^ ^]^ developed an integrated self‐powered controllable transdermal drug delivery system (sc‐TDDS) based on the piezoelectric nanogenerator (PENG) and the conductive microneedle patch (Figure 5f). The sc‐TDDS could release 8.5 ng drugs subcutaneously per electrical stimulation and achieve on‐demand drug delivery. When PENG was driven to produce electrical energy, a linear release profile of the drug was obtained. When PENG stopped working, almost no drug was released from the MNP.

## 4 Closed‐Loop Integrated MN System for Diabetes Management

Closed‐loop systems have emerged as game‐changers, combining glucose monitoring with insulin delivery.^[^ ^3^, ^8^, ^16^ ^]^ These integrated systems promise better glycemic control, reduced episodes of hypoglycemia, and enhanced patient compliance. The application of microneedle technology in diabetes management has been a focal point of research due to the potential advantages microneedles offer over traditional methods of glucose monitoring and insulin delivery. Continuous glucose monitoring (CGM) systems traditionally use a subcutaneous sensor to measure glucose levels in the ISF.^[^ ^13^, ^37^, ^88^ ^]^ Microneedles provide an alternative that's less invasive than the typical CGM sensors. Researchers have developed ultra‐thin microneedles that can painlessly penetrate the skin and monitor glucose levels in real‐time.^[^ ^18^ ^]^ These microneedles are often integrated with wearable devices that alert users to glucose fluctuations. There have been developments in microneedles coated with glucose oxidase, an enzyme that reacts with glucose.^[^ ^3b^ ^]^ This reaction can be measured electrically, providing a potential mechanism for glucose sensing.

**Figure 6:**
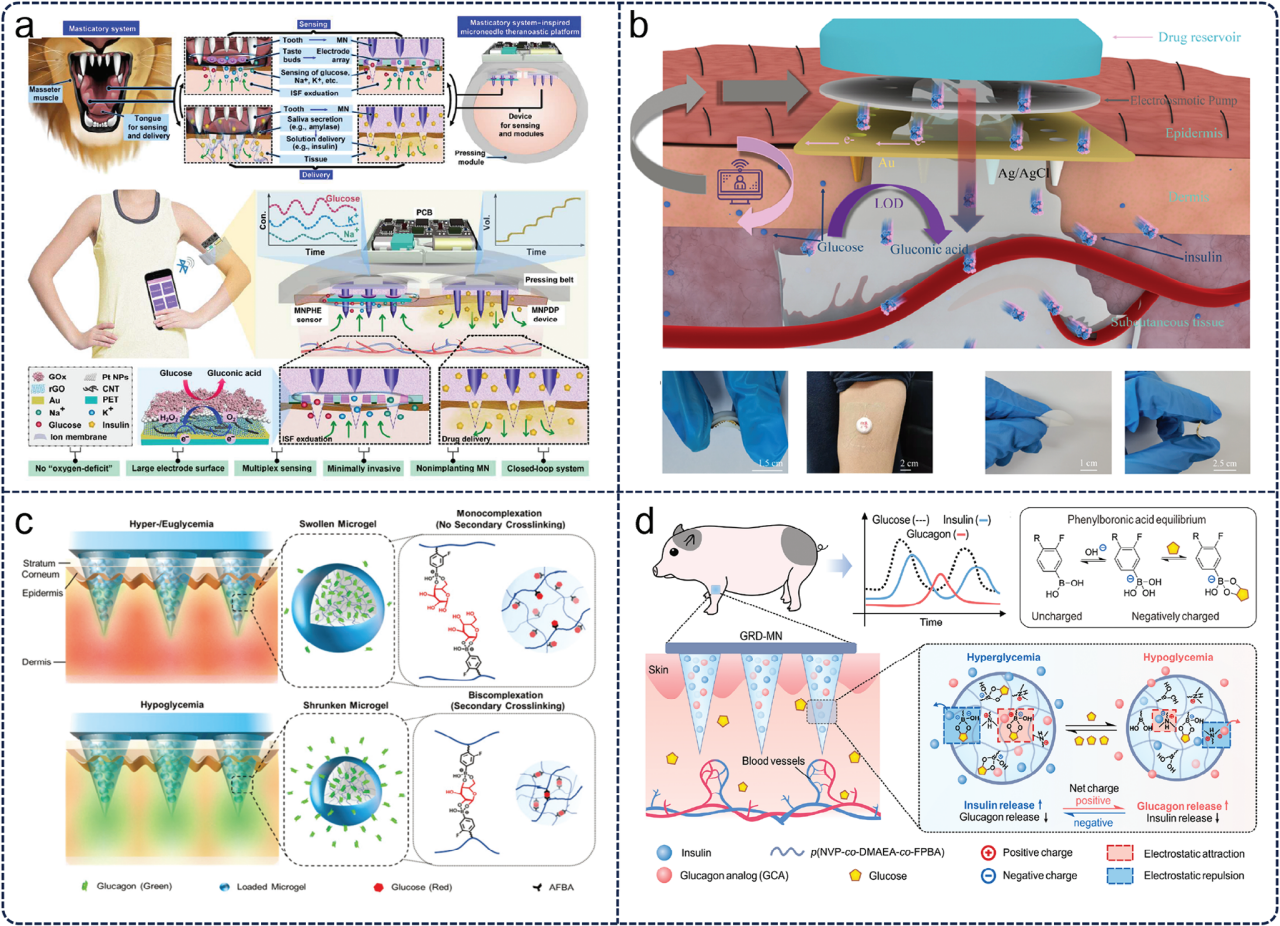
Skin‐integrated MN patch for diabetes management. a) A biomimetic MNs platform for precise diabetes management.^[^ ^89^ ^]^ Copyright 2022, The American Association for the Advancement of Science. b) Closed‐loop diabetic micro‐patch based on hollow biodegradable MN biosensors and electroosmotic pumps.^[^ ^16^ ^]^ Copyright 2022 American Chemical Society. c) Glucose‐responsive MNs patch for hypoglycemia‐triggered natural glucagon delivery.^[^ ^90^ ^]^ Copyright 2019 WILEY‐VCH Verlag GmbH & Co. KGaA, Weinheim. d) Glucose‐responsive MN patch for closed‐loop dual‐hormone delivery.^[^ ^91^ ^]^ Copyright 2022, The American Association for the Advancement of Science.

Microneedles offer a painless or minimally painful method to deliver insulin transdermal, bypassing the need for traditional hypodermic injections. Various designs, including solid, coated, and hollow microneedles, have been explored for insulin delivery. Patches equipped with microneedles made of water‐soluble materials have been developed. These needles dissolve in the skin, releasing insulin over time. For instance, Xie et al.^[^ ^89^ ^]^ introduced a biomimetic microneedle therapeutic platform for intelligent and precise diabetes management (**Figure** **6**a). The platform integrates microcircuits and achieves on‐demand skin puncture through microneedle arrays, thereby allowing ISF to leak out, achieving simultaneous detection of glucose and physiological ions and subcutaneous insulin delivery. Combining microneedle‐based glucose sensing with insulin delivery offers the potential for closed‐loop or “artificial pancreas” systems. Such systems would automatically deliver insulin in response to measured glucose levels, optimizing blood glucose control without constant patient intervention.

The detection and treatment of diabetes mellitus requires regular monitoring of glucose levels to manage and prevent complications. Over time, wearable microneedle patches integrated with sensors, storage units, and wireless communication modules were developed to offer round‐the‐clock monitoring. To achieve miniaturized, painless, low‐cost, and efficient real‐time closed‐loop management of diabetes, Cui et al.^[^ ^16^ ^]^ proposed a novel electrically controlled flexible closed‐loop patch system for continuous diabetes management (Figure 6b). This system integrates hollow degradable microneedles with biosensing devices and electroosmotic pumps to enable real‐time monitoring of glucose levels in ISF and to electrically control the release of insulin based on the monitoring results. Compared to traditional closed‐loop systems, this new patch is miniaturized, painless, low‐cost, and flexible. Additionally, the system can effectively stabilize the blood glucose levels of diabetic rats and is expected to play an important role in the basic research of new closed‐loop devices and the practical application of diabetes management in the future. Diabetic patients frequently require exogenous insulin injections to manage hyperglycemia. However, excessive insulin administration can induce hypoglycemia, a life‐threatening condition characterized by abnormally low blood glucose levels. To mitigate hypoglycemia associated with intensive insulin therapy, Wu et al.^[^ ^90^ ^]^ reported a smart composite microneedle (cMN) patch that releases native glucagon in response to low blood glucose levels (Figure 6c). The cMN patch comprises a photo‐crosslinked methacryloyl hyaluronic acid (MeHA) microneedle array embedded with multifunctional microcapsules. These microcapsules incorporate charged ionic groups that stabilize the encapsulated glucagon and phenylboronic acid groups that enable glucose‐dependent volume changes to facilitate glucagon release. This study represents the first demonstration of a glucose‐responsive glucagon‐releasing microneedle patch for hypoglycemia prevention, highlighting its potential to reduce the risks associated with intensive insulin therapy and improve the quality of life for diabetic patients and their caregivers. Further, Gu et al.^[^ ^91^ ^]^ presented a glucose‐responsive microneedle patch for diabetes treatment that enables closed‐loop delivery of insulin and glucagon, effectively regulating blood glucose levels in mouse and minipig models (Figure 6d). The patch uses phenylboronic acid units to bind with glucose, altering the net charge of the polymeric matrix within the microneedles to adjust the release ratio of insulin and glucagon dynamically. This approach addresses the limitations of existing systems by providing self‐regulating glucagon release, reducing the risk of hypoglycemia, and achieving long‐term glycemic control for over 24 h.

In summary, microneedle technology presents a transformative approach to diabetes management, addressing many of the challenges associated with current treatment and monitoring modalities. The integration of glucose sensing and insulin delivery using microneedles is a promising direction, potentially simplifying and optimizing diabetes care. As research progresses and more products undergo clinical testing, it's anticipated that microneedle‐based solutions will play an increasing role in diabetes management.

## 5 Conclusions and Perspectives

Over the past few decades, MN technology has gained widespread recognition for its successful application in transdermal disease diagnosis and drug delivery. Its unique characteristics, including painless skin penetration, ease of self‐administration, effective therapeutic outcomes, and excellent biocompatibility, have made it a transformative tool in healthcare. This review has highlighted the transformative role of MN technology in the realm of theranostics, which synergistically combines therapeutic and diagnostic functionalities to pioneer advancements in personalized medicine. Initially developed as simple tools for bypassing the skin's primary barrier to drug delivery, microneedles have evolved into sophisticated devices capable of accessing a wealth of biomarkers from ISF, thereby enabling real‐time, non‐invasive health monitoring and management. Some startups are at the forefront of this evolution, driving innovation in microneedle‐based systems. Biolinq, for instance, focuses on continuous glucose monitoring, utilizing microneedles to provide real‐time insights into glucose levels without the need for traditional blood sampling. Similarly, Mylife Technologies is developing microneedles for a range of diagnostic applications, highlighting the versatility and potential of these devices. The versatility of microneedles has been significantly expanded, from solid, traditional structures to innovative dissolvable, and swellable forms, enhancing their application from drug delivery to dynamic, responsive therapeutic systems. Integrated with wearable technology, microneedles contribute to the development of closed‐loop systems that not only monitor physiological states but also adjust therapeutic regimens in real time, reflecting the essence of adaptive, patient‐centered care. These advancements underscore the growing impact of microneedle technology on the future of healthcare, driven by both established research and pioneering startups.

**Figure 7:**
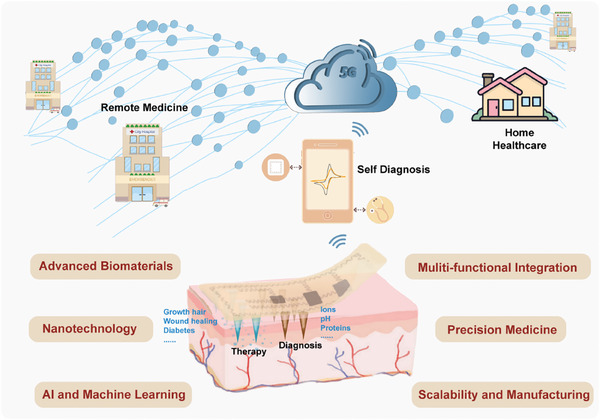
Challenges and perspectives for the next‐generation MNs system.

Looking ahead, MN technology presents immense potential to tackle some of the most pressing challenges in modern healthcare and diagnostics (**Figure** **7**). For example, integrating MN‐based self‐diagnostic and therapeutic systems with 5G technology could pave the way for remote drug delivery and home‐based monitoring, eliminating the need for direct physician presence. However, several critical challenges must be addressed. First, issues of manufacturability and scalability persist, as some fabrication processes are inherently difficult to scale up. Second, balancing mechanical strength with biocompatibility remains a complex tradeoff. While harder materials improve skin penetration, they may cause greater scar tissue formation and stronger immune responses. Durability is another concern, particularly for sensing applications, where long‐term functionality is essential. Furthermore, regulatory hurdles pose significant barriers.

Despite these obstacles, advancements in biomaterials hold promise for the next generation of microneedles. For instance, hydrogel materials with tissue‐like elasticity and strong skin adhesion could enable more efficient and precise drug delivery and diagnostic systems. Innovations in nanotechnology and bioengineering are also expected to expand the capabilities of microneedles, allowing them to deliver a broader range of therapeutics, including novel biologics and small‐molecule drugs, while simultaneously providing molecular protection. In addition, the integrating of digital health technologies such as artificial intelligence (AI) and machine learning with microneedle systems could revolutionize their functionality. Wearable MN biosensors could feature self‐calibrating or calibration‐free designs, leveraging AI to filter and amplify signals, preprocess data to improve signal‐to‐noise ratios, and extract meaningful features from detection signals. The continuous refinement and expansion of microneedle technology will not only drive progress in therapeutic diagnostics but also have a profound impact on global healthcare outcomes. By making personalized and precision medicine more accessible, effective, and convenient, microneedles are set to play a pivotal role in shaping the future of next‐generation healthcare.

## Conflict of Interest

The authors declare no conflict of interest.
